# 2013 Korean Society of Hypertension guidelines for the management of hypertension. Part II—treatments of hypertension

**DOI:** 10.1186/s40885-014-0013-2

**Published:** 2015-04-08

**Authors:** Jinho Shin, Jeong Bae Park, Kwang-il Kim, Ju Han Kim, Dong Heon Yang, Wook Bum Pyun, Young Gweon Kim, Gheun-Ho Kim, Shung Chull Chae, The Guideline Committee of the Korean Society of Hypertension

**Affiliations:** Department of Internal Medicine, Hanyang University College of Medicine, Seoul, Korea; Division of Cardiology, Department of Medicine, Cheil General Hospital, Kwandong University College of Medicine, Seoul, Korea; Department of Internal Medicine, School of Medicine, Seoul National University, Bundang, Korea; Department of Internal Medicine, School of Medicine, Chonnam University, Gwangju, Korea; Division of Cardiology, Department of Internal Medicine, Kyungpook National University School of Medicine, 130 Dongdeok-ro, 700-721 Jung-gu Daegu, Korea; Division of Cardiology, Department of Internal Medicine, Ewha Womans University School of Medicine, Seoul, Korea; Division of Cardiology, Department of Internal Medicine, Dongkuk University School of Medicine, Seoul, Korea

**Keywords:** Antihypertensive treatment, Cardiovascular risk, Guidelines, Hypertension, Lifestyle, Organ damage

## Abstract

Treatment strategies are provided in accordance with the level of global cardiovascular risk, from lifestyle modification in the lower risk group to more comprehensive treatment in the higher risk group. Considering the common trend of combination drug regimen, the choice of the first drug is suggested more liberally according to the physician’s discretion.

## Treatments of hypertension

The purpose of hypertension (HTN) treatment is to prevent cardiovascular disease (CVD) caused by increased blood pressure (BP) and decrease mortality by controlling high BP. In patients who already have established CVD, treatment aims to control BP to prevent progression or recurrence of disease in order to decrease mortality and improve quality of life. HTN treatment provides greater benefit in patients who are at higher risk for CVD. Most clinical studies of HTN have found that lowering systolic blood pressure (SBP) by approximately 10 to 20 mm Hg or diastolic blood pressure (DBP) by approximately 5 to 10 mm Hg can reduce the occurrence of stroke by 30% to 40% and that of ischemic heart disease by 15% to 20% [1]. Because most clinical studies were performed over a relatively short period, the benefits of HTN treatment over a period of 5 years or more seem to be much more pronounced than the treatment efficacy observed in clinical studies. The benefits of HTN treatment are not affected by sex or age and are also similar for the treatment of systolic HTN in the elderly. HTN treatment was found to be the most cost-effective intervention for prevention of CVD.

## Strategy for hypertension treatment

**Figure 1:**
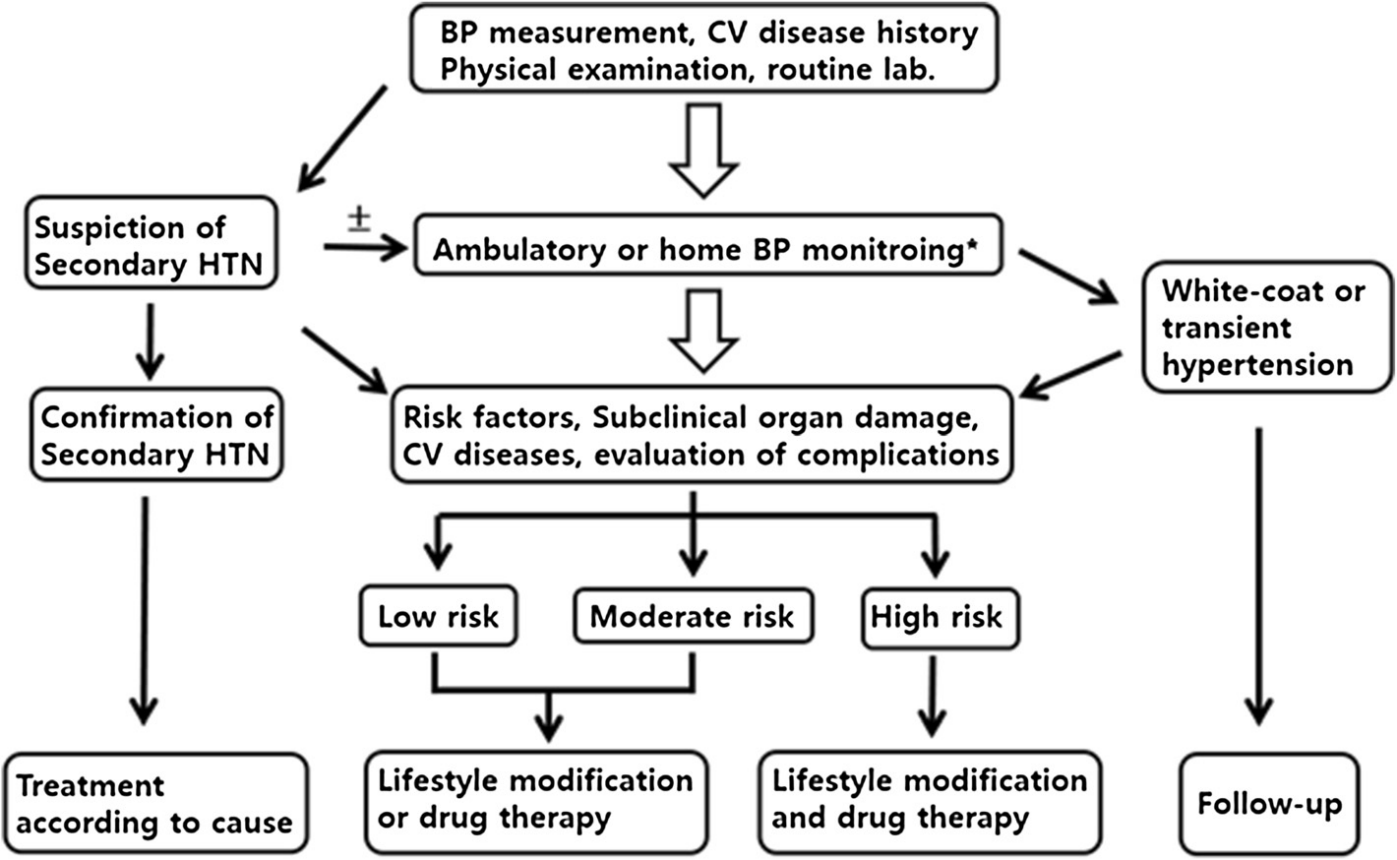
**Treatment strategies for hypertension.** *BP* blood pressure; *CV* cardiovascular; *HTN* hypertension. ± Optional. ^*^Recommended test. ^†^In the moderate to high risk group, drug therapy can be considered for stage 2 hypertension patients.

If a patient is already known to have high BP, the diagnosis of HTN must be confirmed prior to treatment by measuring the out-of-clinic BP, such as at home or using 24-h monitoring. Measurement of out-of-clinic BP helps not only to obtain a more accurate diagnosis but also to determine the appropriate treatment for the patient and to increase treatment compliance. If HTN is diagnosed, the risk factors for CVD, associated diseases, and existence of hypertensive complications should be investigated (Figure 1). Patients with white coat HTN, which is defined as high BP in but not out of the clinic, must be followed up periodically at 3- to 6-month intervals because their risk of CVD increases over time. HTN treatment must include nondrug therapy (such as lifestyle modification) concomitant with drug therapy. The initiation of drug therapy needs to be considered and determined on the basis of not only the BP level but also the presence of risk factors for CVD and evidence of damage to target organs. Drug therapy may be used in patients with a BP of 140/90 mm Hg or higher regardless of the existence of other risk factors or associated diseases. The quality of life of patients with HTN can be affected by physical and psychological problems caused by HTN, the main and side effects of the drug, and the relationship between the patient and physician. Adequate communication and provision of information can decrease the dosage and frequency of medication used, which increases patient compliance, improves the BP control rate, and promotes continuous treatment.

## Initiation of hypertension treatment

### Prehypertension stage II

Cardiovascular (CV) mortality caused by HTN increases twofold for each 20-mm Hg increase in SBP or 10-mm Hg increase in DBP over the baseline level of 115/75 mm Hg [1,2]. Therefore, in patients with BP over 120/80 mm Hg, nondrug therapy is recommended to prevent the occurrence of HTN and CV events. Initiation of drug therapy in patients with stage 2 prehypertension can delay the progression to HTN [3,4], but there is little evidence for the effectiveness of early intervention in most clinical studies [5]. Initiation of drug therapy during stage 2 prehypertension showed no consistent benefit in patients with prediabetes [6,7], DM [8], stroke [9], or coronary artery disease; [10] therefore, the cost-benefit ratio should be considered in the decision to use drug therapy (Table 1).

**Table 1:** **Treatment for hypertension according to the risk**

**Risk factor**	**Blood pressure (mm Hg)**		
	**Stage 2 prehypertension (130–139/85–89)**	**Stage 1 hypertension (140–159/90–99)**	**Stage 2 hypertension (≥160/100)**
Risk factors 0	Lifestyle modification	Lifestyle modification^a^ or drug therapy	Lifestyle modification or drug therapy^†^
Risk factors 1–2 other than DM	Lifestyle modification	Lifestyle modification^a^ or drug therapy	Lifestyle modification and drug therapy
Risk factors ≥3, subclinical organ damage	Lifestyle modification	Lifestyle modification and drug therapy	Lifestyle modification and drug therapy
DM, cardiovascular disease, chronic kidney disease	Lifestyle modification or drug therapy^b^	Lifestyle modification and drug therapy	Lifestyle modification and drug therapy

*DM* diabetic mellitus.

^a^Lifestyle modification is carried within several weeks to 3 months. ^†^Drug therapy may be begun immediately according to the height of BPs.

^b^Drug therapy may be begun as target blood pressure determined.

### Hypertension stage I

Patients with stage 1 HTN without other risk factors are in the low-risk group and would not be expected to obtain a greater benefit from treatment [11]. However, their overall risk will increase over time, and the window in which treatment could reverse progression might be missed. Modern drugs are generally inexpensive and safe, and drug therapy has been found to be cost-effective given that patients generally fail to accomplish lifestyle changes. Drug therapy is recommended only after measurement of the out-of-clinic BP in order to exclude the possibility of white coat HTN [12,13]. The effect of medical therapy on white coat HTN has not yet been proven; however, as white coat HTN poses increased metabolic risk and risk for CV events over the long term, lifestyle modification is recommended at first, and patients should be observed periodically for development of persistent HTN. Drug therapy should be instituted immediately in patients with high-risk stage I HTN [14,15].

### Hypertension stage II

According to most randomized clinical trials, immediate drug therapy is warranted in patients with a BP of ≥160/100 mm Hg due to the noticeable effect of treatment [5,14,15].

### Hypertension in the elderly

The effect of drug therapy against HTN is clear irrespective of age [16]. Drug therapy can be initiated in elderly patients when the SBP is 160 mm Hg or higher; consistent drug therapy can be considered if the SBP is 140 to 159 mm Hg and the patient tolerates the drug well.

## Target blood pressure in the treatment of hypertension

As shown in Table 2, except under specific circumstances, the target BP is generally an SBP of less than 140 mm Hg and a DBP of less than 90 mm Hg [5,17,18].

**Table 2:** **Target blood pressures in hypertension treatment**

**Situations**	**Systolic blood pressure (mm Hg)**	**Diastolic blood pressure (mm Hg)**
Uncomplicated hypertension	140	90
Elderly	140–150	90
Diabetes mellitus	140	85
Stroke	140	90
Coronary artery disease	140	90
Chronic kidney disease		
Without albuminuria^a^	140	90
With albuminuria	130	80

^a^Microalbuminuria or macroalbuminuria.

### Hypertension in the elderly

The effects of decreasing BP in elderly patients with HTN are clear, but it is difficult to lower BP to less than 140 mm Hg in such patients [19], and there is no difference in prognosis between BP targets of 140 and 150 mm Hg. Therefore, the target SBP is approximately 140 to 150 mm Hg with a DBP that is not excessively low, i.e., less than approximately 60 mm Hg [20,21].

### Hypertension in patients with diabetes

Reduction of BP in patients with HTN and diabetes is very important for reducing CV complications [22]. Few studies have shown reduction of SBP to below 130 mm Hg. Even reduction of SBP to less than 120 mm Hg did not demonstrate any additional preventive effect on CVD but rather showed a deleterious effect on renal function; therefore, the recommended target BP is an SBP below 140 mm Hg [23] and a DBP below 85 mm Hg [24].

### Hypertension in patients with stroke

HTN is a most important causative risk factor for stroke. HTN treatment can reduce the recurrence of stroke and CV events [25-27], but there are no distinct benefits from reducing SBP to below 130 mm Hg [28]. In particular, a recent clinical study in patients with cerebral infarction showed no additional benefit from controlling SBP below 140 mm Hg [29]. Considering the clinical studies to date, a target SBP below 140 mm Hg is recommended in patients with stroke.

### Hypertension in patients with coronary artery disease

Reduction of SBP to below 130 mmHg shows no consistent prevention of CVD in patients with HTN and coronary artery disease [30-32]. Therefore, a target SBP of below 140 mm Hg is recommended.

### Hypertension in patients with chronic kidney disease

The major purpose of controlling BP in patients with chronic kidney disease (CKD) is to prevent deterioration of renal function and reduce the occurrence of CVD. Further control of SBP to below 140 mm Hg has shown no additional benefit in patients with HTN and CKD without diabetes [33-35]. However, the data on the goal of treatment in patients with HTN and CKD with diabetes is even more insufficient [36,37]. Meta-analysis has not proven that a target BP of less than 140 mm Hg is any more effective at preventing cardiac and renal events [23,38,39]. Therefore, on the basis of recent clinical data, a target SBP of less than 140 mm Hg is recommended regardless of the presence of diabetes. However, a target SBP below 130 mm Hg can be recommended in patients with HTN with prominent albuminuria [40].

### Low threshold of hypertension treatment

As BP increases, the CV risk also increases, whereas as BP decreases, the risk of the occurrence of a CV event decreases to some extent. There is insufficient clinical data to prove the J-curve hypothesis (a hypothesis that excessive lowering of SBP and DBP will increase rather than decrease CV events and mortality), but *post hoc* analysis of clinical studies suggests the possibility of such a J-curve effect and a pathophysiological detriment of excessively low BP [41]. Therefore, it is not recommended to target BP too low, but additional studies are needed to determine how low a BP is desirable.

## Nondrug therapy and lifestyle modification

Nondrug therapy or lifestyle modification, such as adoption of a healthy diet, increasing exercise, smoking cessation, and moderation of alcohol intake, has shown great ability to lower BP and is important in all patients with HTN. Even in patients with prehypertension, nondrug therapy is strongly recommended to prevent progression to HTN (Table 3). Healthy lifestyle habits have almost the same BP-decreasing effect as approximately one dose of anti-HTN drug [42]. Furthermore, in patients with HTN who are using medication, adding lifestyle modification can reduce the dose and frequency of medication used, maximize the effect of the drug, and reduce side effects. Lifestyle improvement also has other beneficial effects on CV risk in addition to the lowering of BP. Clinicians should remain aware that it is difficult to maintain lifestyle modifications long-term or to achieve a target BP for the HTN in stage II or higher even at best. Therefore, the clinician should provide encouragement to continue lifestyle modifications while also educating the patient in their limitations. In addition, because adopting several types of lifestyle modification rather than one alone maximizes the effects, a simultaneous approach is recommended to meet the goal of minimizing CVD.

**Table 3:** **Blood pressure reduction by lifestyle modification**

**Lifestyle modification**	**BP reduction (systolic/diastolic BP, mm Hg)**	**Recommendation**
Restriction of salt intake	−5.1/−2.7	Less than 6 g of salt per day
Body weight reduction	−1.1/−0.9	Each reduction of 1 kg
Moderation in drink	−3.9/−2.4	Less than two glasses per day
Exercise	−4.9/−3.7	30–50 min per day for more than 5 days in a week
Diet control	−11.4/−5.5	Vegetables-based healthy diet habit

*BP* blood pressure.

### Restriction of salt intake

According to the 2010 KNHANES data, it is estimated that Korean consume 12.2 g (4.9 g sodium) of salt daily, which is a higher amount than western (10 g) or Japanese (10.7 g) populations [43]. Salt intake can increase the risk for CV events because of the association of central hemodynamics with the estimated 24-h urinary sodium in patients with HTN [44]. Halving the daily salt intake of 10.5 g will decrease SBP by 4 to 6 mm Hg [45,46]. However, there have been variable and confusing reports about the relationship between salt restriction and CV events [47-50]. Despite the absence of randomized large-scale trials, some reports of a J-curve phenomenon for salt intake and CV events, and lack of Korean data, there is no clear evidence that salt restriction is harmful, especially among Koreans with their high-salt intake. Therefore, we must recommend decreasing salt intake in accordance with other guidelines [51].

The daily recommended amount of salt is less than 6 g (sodium [g] × 2.5). Salt reduction has many benefits, including lowering of BP and reducing the need for diuretics, which cause detrimental urinary loss of potassium and calcium. The avoidance of urinary calcium loss prevents the development of osteoporosis and renal calculi.

Sensitivity to salt tends to be higher in patients who are elderly, obese, or have diabetes or family members with HTN. Greater salt sensitivity means a greater reduction in BP in response to salt restriction. The dietary habits must be modified. Some recommendations are not to put additional salt on the table during the meal and to avoid high-salt processed foodstuffs. Some frequently eaten foods, such as kimchi, stew, soup, salted fermented seafood, instant ramen, and dry bar snacks containing meat and fish, are very salty. When cooking foods, natural ingredients should be used instead of synthetic flavoring agents. The patient should not only reduce absolute salt intake but also try to consume more low-salt foods.

### Weight reduction

HTN is closely related to obesity, and weight reduction decreases BP. Central obesity in particular is closely associated with HTN, dyslipidemia, diabetes, and CV death. In a patient heavier than 110% of ideal body weight, weight reduction of only 5 kg can decrease BP. The beneficial effects of weight reduction are higher in patients with diabetes, dyslipidemia, and left ventricular hypertrophy (LVH). The combination of weight reduction with exercise, moderation of alcohol consumption, and reduction of salt intake has synergistic effects on BP. The recommended initial goal of weight reduction is 4 to 5 kg, with an additional 5 kg reduction after the initial goal has been achieved.

The ideal body mass index (BMI) (weight [kg]/height [m]^2^) varies among reports and according to nationality. A collaborative analysis reported that BMI either above or below the apparent optimum of approximately 22.5 to 25 kg/m^2^ is itself a strong predictor of overall mortality [52]. Another meta-analysis found that both overweight and grade 1 obesity were associated with significantly lower all-cause mortality [53]. A report on data from 1.2 million Koreans revealed that the risk of death from any cause was lowest among patients with a BMI of 23.0 to 24.9 kg/m^2^ and recommended a BMI of less than 25 kg/m^2^ [54]. Unfortunately, there are no Korean-specific data on appropriate waist circumference. A waist circumference of less than 90 cm for men and 80 cm for women is recommended for Asian individuals [55].

The recommendations for weight reduction are to eat breakfast every morning, eat slowly, and avoid a high-carbohydrate diet, alcohol, snacks such as bread and cookies, and sweetened beverages. A high-fiber diet is recommended, and a high-fat diet including food fried with oil is prohibited. Patients should try to eat as many fruits and vegetables as possible and to avoid meals containing large amounts of cholesterol and saturated fatty acids.

### Moderation of alcohol consumption

BP tends to increase in patients who drink excessive amounts of alcohol, and such patients are also resistant to antihypertensive drugs. An appropriate moderate daily amount of alcohol is less than 20 to 30 g for men or 10 to 20 g for women. A man or woman with lower-than-average body weight is more sensitive to alcohol and is therefore permitted half of the recommended amount. Heavy drinkers should be warned that they are high risk for stroke. One bottle of beer (720 mL), one glass of wine (range, 200 to 300 mL), one glass of sake (200 mL), two shots of whisky (60 mL), or two to three glasses of soju corresponds to 30 g of alcohol.

### Exercise

The benefits of regular exercise are lowering of BP, improvement of cardiopulmonary function, reduction of body weight, improvement of the lipid profile (including elevation of HDL cholesterol), and reduction of emotional stress. Aerobic exercises such as brisk walking, jogging, bicycling, swimming, jumping rope, playing tennis, and aerobic dancing are recommended for patients with HTN. The appropriate intensity of exercise is 60% to 80% of the maximal heart rate (220—age in years). Such exercise should be performed five to seven times per week. Aerobic exercise should begin at low intensity for 10 to 20 min and then increase to appropriate intensity for another 30 to 60 min. Every exercise session should start with 5 min of warm-up exercise and end with 5 min of cool-down exercise. In addition to aerobic exercise, isometric exercise such as weight lifting can reduce BP, produce metabolic benefits, and increase muscle power. Isometric exercise is recommended two to three times per week [56]. Isotonic exercise such as lifting a heavy object (anaerobic metabolism) can increase BP and may be dangerous in patients with poorly controlled HTN. Most patients with uncomplicated HTN can begin regular exercise without an initial evaluation and increase the duration and intensity to appropriate levels as possible. However, patients with known CVDs or other risk factors are recommended to start the exercise only after complete evaluation by an exercise consultant and to follow a program.

### Smoking cessation

During smoking, the BP increases temporarily in response to nicotine. Among patients with white coat HTN, smokers maintain a higher daytime ambulatory SBP than do nonsmokers with a similar office BP [57]. Smoking, like HTN, is a powerful risk factor for CVD [58], and CV events are inevitable in patients who continue smoking regardless of BP control. Second-hand smoking is also harmful. Smoking cessation should be advised at every opportunity. Low-nicotine-containing replacement materials do not increase BP and can be recommended in combination with behavior therapy. During smoking cessation, regular exercise and diet therapy should be emphasized in order to prevent weight gain.

### Healthy diet management

BP is lower in vegetarians than in people who mainly eat meat, and maintaining a vegetarian diet can reduce BP. The BP-lowering effect results not from decreasing the intake of animal protein but from increasing the intake of vegetables and fruits in combination with decreasing the intake of saturated fatty acids. In a study in elderly people, BP decreased by 3/1 mm Hg when intake of vegetables and fruits was increased alone but by 6/3 mmHg when it was combined with a decrease in fat intake [59-61]. In patients with HTN, a combined diet with greater intake of calcium, magnesium, and potassium decreased BP by an additional 11/6 mm Hg [59,61,62]. A regular diet composed mainly of fish reduces BP and improves the lipid profile in obese patients with HTN. The Dietary Approaches to Stop HTN (DASH) diet, which is rich in vegetables, fruits, and fish and low in fat, is recommended for patients with HTN [63].

### Others

Caffeine from various foods rapidly increases BP, but the effect does not progress to HTN because tolerance to caffeine develops. Emotional stress increases both BP and the risk for CVD, making the control of emotional stress important for the management and patient compliance of HTN. Further studies are required to examine the long-term effects of stress control on HTN and CVD. The effectiveness of various methods of stress management, such as relaxation and biofeedback, for the management of HTN remains uncertain. There is still no clear evidence for the effects of micronutrients, calcium, magnesium, and supplementary fiber on BP.

## Pharmacological therapy

The occurrence of CV events in patients with HTN can be decreased by reducing the BP. Currently available antihypertensive drugs are more effective than placebo for prevention of CVD. This preventive effect is relatively larger for stroke than for coronary artery disease. The extent to which CV events are reduced depends on the degree of BP reduction. All major classes of antihypertensive drugs, including beta-blockers and diuretics, are suitable for first-line treatment. However, the individual drug should be prescribed with consideration of the patient’s individual situation, including age, comorbidities, and possible adverse effects. Simplifying the medication schedule, careful monitoring of the adverse effects, and checking the BP at home are useful for improving patient compliance and making the patient an active participant in the treatment.

## Strategies for prescription of antihypertensive drugs

### Principles of drug selection

For reduction of long-term CV morbidity and mortality, it is essential to control most of the modifiable risk factors and to reduce the BP to less than 140/90 mm Hg [40]. Drug therapy is initiated at a low dose to avoid adverse effects. The preferred drugs are long-acting and can be taken only once a day [64]. Drugs with a high trough/peak ratio (>0.5) are helpful for improving compliance and to maintain a stable BP with minimal variability [65]. If it is impossible to control BP with once-daily dosing, a twice-daily schedule is an alternative option. Angiotensin-converting enzyme (ACE) inhibitors, angiotensin receptor blockers (ARBs), calcium antagonists, beta-blockers, and diuretics are all suitable for initiation of antihypertensive treatment. The indications, contraindications, comorbidities, and presence of asymptomatic organ damage should all be considered in the choice of drug (Table 4). There is no uniform consensus on the role of beta-blockers in elderly patients with HTN, so prescription of beta-blockers in the elderly should be limited to special circumstances. Beta-blockers should also be used with care in patients at high risk for diabetes because in combination with diuretics they can increase the risk of new onset of diabetes [40]. In patients with BP higher than 160/100 mm Hg or more than 20/10 mm Hg above the target BP, two drugs can be prescribed in combination to maximize the antihypertensive effect and achieve rapid BP control [40]. Fixed-dose single pills have multiple benefits, including maximizing reduction of BP, minimizing adverse effects, increasing compliance, and preventing CVD and target organ damage [40].

**Table 4:** **Compelling indications for choosing the antihypertensive drugs** [40,66]

	**Angiotensin-converting enzyme inhibitors/angiotensin receptor blockers**	**Beta-blockers**	**Calcium antagonists**	**Diuretics**
Congestive heart failure	O	O		O
Left ventricular hypertrophy	O		O	
Coronary artery disease	O	O		
Diabetic nephropathy	O			
Stroke	O		O	O
Elderly, isolated systolic hypertension	O		O	O
Post-myocardial infarction	O	O		
Prevention of atrial fibrillation	O			
Diabetes	O			

### Selection of drugs

It is reasonable to choose drugs according to the patient’s comorbidities and clinical characteristics rather than his or her BP level. There are five available classes of first-line drugs with proven BP-lowering effects, safety, and acceptable adverse effects according to multiple studies. They are: 1) ACE inhibitors or ARBs, 2) beta-blockers, 3) calcium antagonists, 4) diuretics such as hydrochlorothiazide, chlorthalidone, or indapamide, and 5) other drugs (loop diuretics, aldosterone antagonists, alpha-blockers, and direct vasodilators). All reduce BP to a similar extent when the dose has been adjusted. However, there might be individual differences in BP lowering, adverse effects, and long-term CV events, making it very important to choose the appropriate drugs according to the patient’s combined risk factors and comorbidities (Table 5). No antihypertensive drug is inherently superior, and the drugs most appropriate for the individual patient should be preferred (Table 5).

**Table 5:** **Indications and contraindications of antihypertensive drugs**

	**Absolute indications**	**Relative indications**	**Need cautions**	**Absolute contraindications**
Angiotensin-converting enzyme inhibitors/angiotensin receptor blockers	CHF, diabetic nephropathy		Renal artery stenosis, hyperkalemia	Pregnancy, angioedema
Beta-blockers	Ischemic heart disease, myocardial infarction	Tachyarrhythmia	High blood glucose, peripheral artery disease	Asthma, severe and symptomatic bradyarrhythmia
Calcium antagonists	Elderly hypertension, isolate systolic hypertension, ischemic heart disease (non-dihydropyridine calcium antagonists)		CHF	Severe and symptomatic bradyarrhythmia
Diuretics	CHF, isolated systolic hypertension		High blood glucose	Gout hypokalemia

*CHF* congestive heart failure.

## Classes of antihypertensive drugs

### Diuretics

Diuretics decrease BP initially by reducing reabsorption of sodium in the renal distal convoluted tubules and later by decreasing peripheral vascular resistance. High-dose thiazide-derivative diuretics can induce hypokalemia, glucose intolerance, hyperuricemia, arrhythmia, and adverse lipid metabolism, but low doses rarely have these effects. For decreasing BP, the dose can be increased to 25 mg a day. Combination of diuretics with beta-blockers is not recommended in patients with obesity or high risk for diabetes because of adverse effects such as new-onset diabetes and adverse lipid metabolism. No specific thiazide diuretic is recommended over the others because there has been no study comparing them directly, although chlorthalidone and indapamide are reported to be most effective for lowering BP [67]. As thiazide diuretics must frequently be administered at high doses to achieve optimal BP control, and because such high doses increase the adverse effects, thiazide-like diuretics may be preferred to avoid high dosage and/or reduce metabolic derangement. Spironolactone is proven effective in patients with heart failure and can also be considered at low doses (range, 20 to 50 mg) for treatment of resistant HTN.

### Beta-blockers

Selective beta-1 blockers are recommended for patients with HTN in combination with angina pectoris, myocardial infarction, or tachycardia. Beta-blockers are also effective in younger patients who have higher heart rates [40]. However, they should be used with caution in patients with asthma, chronic obstructive pulmonary disease, second- or third-degree atrioventricular block, or peripheral vascular disease [40].

Beta-blockers can have adverse effects on blood glucose and lipid metabolism and should therefore be used cautiously in elderly patients or patients with elevated blood sugar, diabetes, or metabolic syndrome [68]. They should also be used carefully in patients with variant angina because they can worsen symptoms. [69] Because atenolol is inferior for stroke prevention, first-line therapy is not recommended for elderly patients with HTN [70]. Concomitant use of beta-blockers and diuretics will increase the incidence of diabetes and should therefore be avoided in patients at high risk for developing diabetes [68]. Vasodilatory beta-blockers might have different effects than atenolol, but no comparative study has yet been performed [71,72].

### Calcium antagonists

Long-acting calcium antagonists are preferable to short-acting calcium antagonists, which can cause tachycardia and increase cardiac workload. Because calcium antagonists have a vasodilatory effect on the coronary artery, they are highly effective in patients with stable angina or variant angina, which is caused by coronary artery spasm. They are also effective for slowing the progression of carotid atherosclerosis and reducing cardiac hypertrophy [73]. The non-dihydropyridine calcium antagonists, verapamil and diltiazem, are effective after myocardial infarction because they do not produce reflex tachycardia. They are also effective in patients with hypertrophic cardiomyopathy because they improve diastolic filling. The common side effects of dihydropyridine calcium antagonists are tachycardia, ankle edema, headache, and facial flushing. Non-dihydropyridine calcium antagonists may cause constipation, conduction delay, and decreased myocardial contractility and should therefore be prescribed cautiously to patients with systolic heart failure or heart block. In addition, special caution is needed when administering them in combination with beta-blockers in elderly patients.

### Angiotensin-converting enzyme inhibitors/angiotensin receptor blockers

ACE inhibitors/angiotensin receptor blockers reduce mortality in patients with heart failure and help to inhibit the progression of renal disease. They also help to prevent LVH and atherosclerosis but have little effect on blood glucose or lipid metabolism [74]. In addition, they can improve vascular endothelial cell function and promote revascularization. However, they can cause a hypotensive response in dehydrated or elderly patients [75]. When administered to a patient with bilateral renal artery stenosis, they can have adverse effects such as severe hypotension and deterioration of renal function [75]. The serum creatinine level may increase within the first 2 months after the start of treatment. However, there is no need to discontinue the drug unless the serum creatinine increases to less than 30% rise than the baseline creatinine level or unless serum potassium is 5.5 mEq/L or higher [76]. Care should be taken in patients with a serum creatinine level higher than 3.0 mg/dL [77]. The blood potassium level and renal function should be checked before and within 1 to 2 weeks after administration of the drug and then again 3 or 6 months later. ACE inhibitors inhibit bradykinin degradation and can thus cause a dry cough, but this resolves within a few days to a few weeks after stopping the medication. Dry cough is more common in women and nonsmokers. Angiotensin receptor blockers have no effect on bradykinin and therefore rarely cause cough. ACE inhibitors/angiotensin receptor blockers are contraindicated in pregnant women because of their teratogenic effects on the fetus.

### Others

Alpha-blockers can alleviate urinary symptoms in patients with prostate enlargement and also improve the metabolism of sugars and lipids. However, they can cause orthostatic hypotension and are associated with worsening of heart failure. Agents that act on the central nervous system, such as clonidine, methyldopa, and reserpine, have many side effects and are therefore not recommended as first-line drugs. Renin inhibitors have been developed and used in other countries but have not yet been introduced in Korea. Renin inhibitors significantly reduced BP and proteinuria when used alone or in combination with diuretics. However, aliskiren has not been proven to improve the prognosis of patients with CVD. Methyldopa is still preferred for the treatment of HTN in pregnant women but is not the first choice because of its side effects. Hydralazine is a vasodilator that is relatively safe for pregnant women with HTN.

## Combination therapy

**Figure 2:**
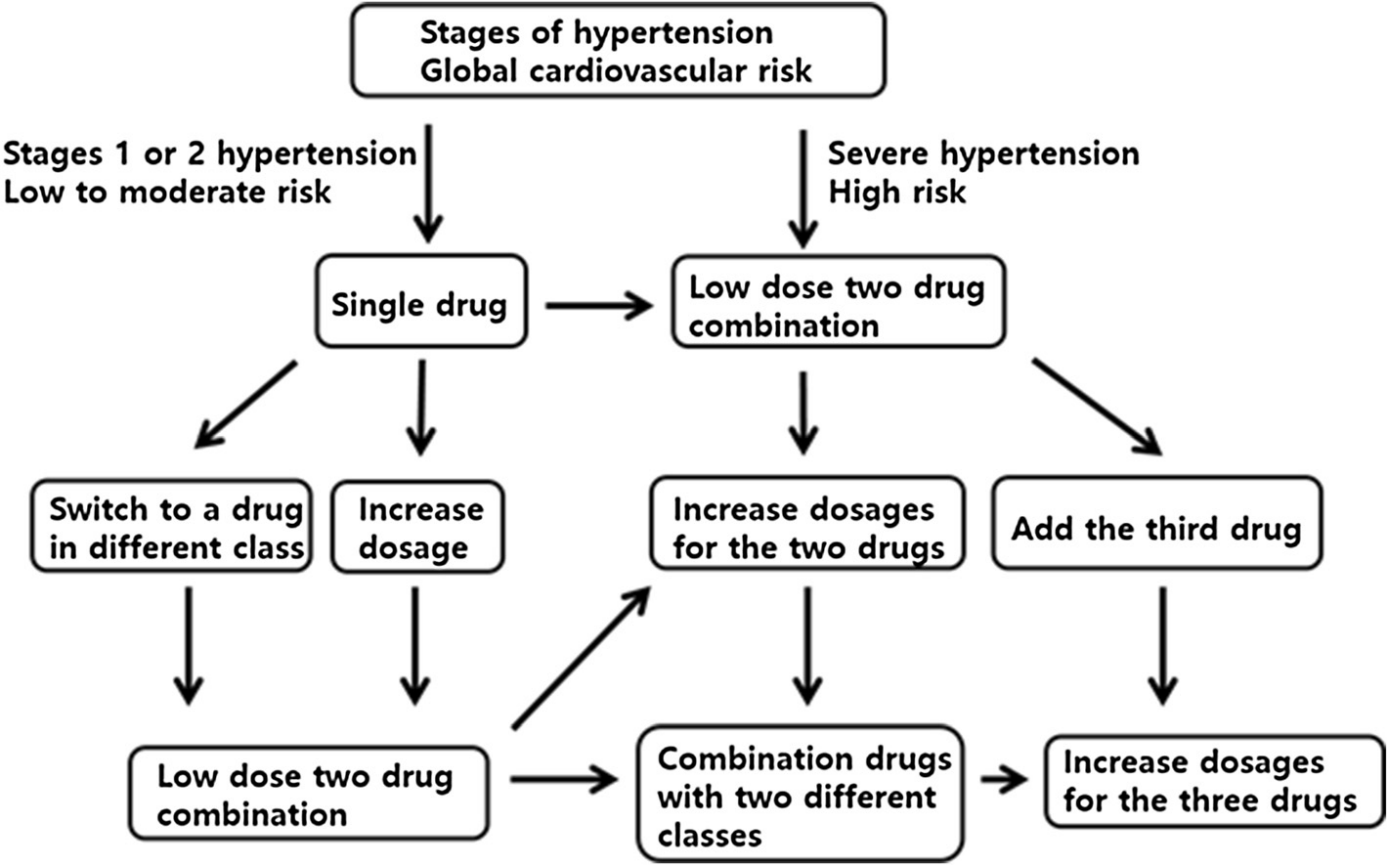
**Choice of single drug or combination drugs according to the level of blood pressure and the global cardiovascular risk.**

More than 2/3 of patients with HTN need drugs from more than two drug classes with different mechanisms to achieve control of HTN. Combination therapy is particularly helpful for patients receiving prolonged BP treatment, high-risk patients, and patients with a low target BP. If the first drug used is not effective for BP control, then a drug of another class should be tried. If the efficacy is insufficient, the dose should be increased or another drug added. However, it is recommended to combine two different drugs in smaller doses rather than to increase the dosage of one drug because such low-dose combinations lower BP more effectively and improve the compliance while decreasing the adverse effects (Figure 2) [78].

**Figure 3:**
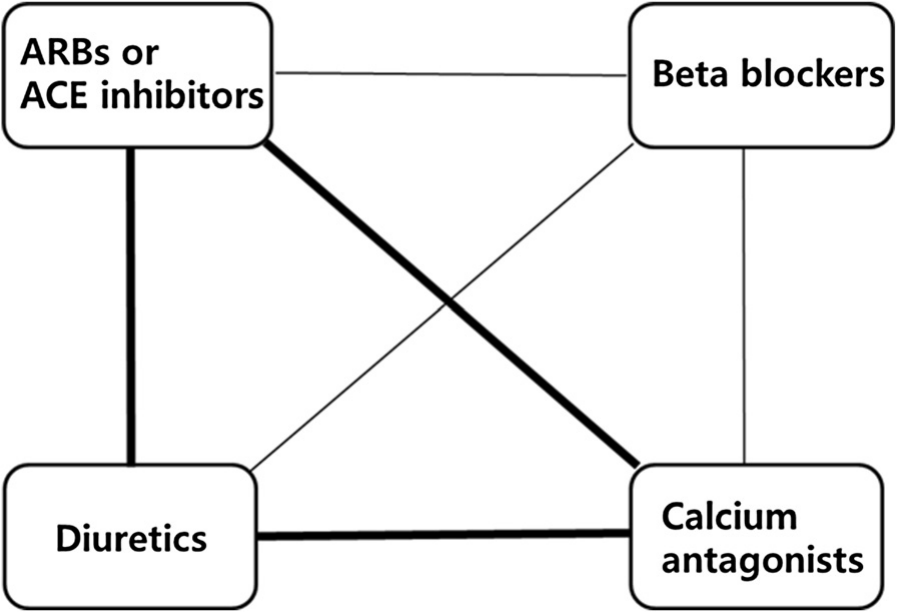
**Recommended combination therapy, thick lines; preferred combination, thin line; feasible combination.** *ARB* angiotensin receptor blocker, *ACE* angiotensin-converting enzyme.

If BP is not controlled with a single drug, two drugs should be combined for BP control. Combination therapy is more effective than single-drug therapy at a higher dose [78]. However, it has not been fully evaluated which combination is best. Combination therapy chosen from the renin-angiotensin system inhibitors, calcium antagonists, and diuretics is recommended first because it has shown relatively good results [17,79,80], but beta-blockers can also be combined with drugs of other classes (Figure 3). However, the combination of beta-blockers and diuretics can increase the incidence of diabetes and metabolic disorders and thus requires periodic monitoring. Combination therapy with angiotensin-receptor blockers and ACE inhibitors may be slightly more effective for reducing proteinuria but increases the risk for end-stage renal failure, stroke, and other CVD [81-83].

## Resistant hypertension

Resistant HTN is defined as BP that cannot be controlled (BP ≥ 140/90 mm Hg) despite treatment with more than three different classes of antihypertensive drugs, including diuretics. The prevalence of resistant HTN is reported to be 5% to 30% in other countries. However, considering the frequency of pseudo-resistant HTN, the prevalence of true resistant HTN is assumed to be less than 10% [40]. Patients with resistant HTN are at much higher risk for complications such as CVD and kidney disease [84].

Among the wide range of causes of resistant HTN (Table 6), noncompliance is the most common. In addition, medications taken for relief of cold symptoms, nonsteroidal anti-inflammatory drugs, adrenal cortical steroids, birth control pills, excessive salt intake, and excessive alcohol consumption can also cause resistant HTN. If diuretics have not been included in the regimen, volume overload can cause resistant HTN. Finally, secondary HTN can cause resistant HTN. To diagnose resistant HTN, treatment compliance should be confirmed and then the home BP or ambulatory BP checked in order to exclude white coat HTN. If BP cannot be controlled despite the use of effective doses of three different classes of drug, then the dose of diuretics should be increased, or amiloride added, or thiazide diuretics changed to loop diuretics in patients with renal impairment. However, most patients with resistant HTN require a different mechanism for BP control, and the fourth drug added should be spironolactone or an alpha-blocker such as doxazosin [85-88].

**Table 6:** **Differential diagnosis of uncontrolled hypertension**

**Diagnoses**	**Causes**
Pseudo-resistant hypertension	
	Poor compliance
	Wrong cuff use
	Using too small cuff
	White coat hypertension
	Calcified vessel in the elderly (pseudohypertension)
Resistant hypertension	
	Lifestyle factors: severe weight gain, heavy or binge drinking, excess salt intake
	Medication: cold remedies, nonsteroidal anti-inflammatory drugs, corticosteroid, cyclosporine/tacrolimus, erythropoietin, cocaine, herbal licorice
	Secondary hypertension
	Sleep apnea syndrome
	Volume expansion by renal diseases
	Vascular damage or stiffness
	Prescription of antihypertensive drugs: insufficient dose, wrong use of diuretics, ineffective combination, drug interaction

### Renal denervation

Bilateral destruction of the renal sympathetic nerves that course along the renal artery by the use of radiofrequency ablation catheters is an increasingly popular nondrug therapeutic approach to HTN. Reduction of BP lasts for more than a year after the procedure, and the BP-reducing effect was reported to be maintained for another 3 years of follow-up [89].

The treatment itself has no significant complications and can therefore be used in patients with resistant HTN. However, there is insufficient short-term and long-term evidence for the efficacy of renal denervation [90]. Carotid stimulation is also reported to be effective for lowering BP in patients with resistant HTN; however, there is very little randomized blind study data supporting its efficacy [91].

## Reduction or discontinuation of antihypertensive medications

In patients whose BP has been well controlled for years, the dose of antihypertensive drug can be reduced. Afterwards, the BP should be checked regularly, and the continued practice of lifestyle modifications monitored [40].

## Other drug treatments

The goal of antihypertensive therapy is to reduce the overall CV risk in patients who have other risk factors such as diabetes, dyslipidemia, coronary artery disease, stroke, and CKD. Accordingly, these other risk factors should be treated at the same time.

### Antiplatelet therapy

Aspirin administration was shown to produce an absolute benefit for the secondary prevention of CVD in patients with HTN [92]. However, the role of aspirin for secondary prevention remains a matter of debate.

Low-dose aspirin (100 mg) can be prescribed to patients in high-risk groups in order to reduce the risk of CVD [92,93]. Antiplatelet agents should be administered after the BP is controlled, and patients should be checked periodically for gastrointestinal bleeding.

### Lipid-lowering agents

Lipid-lowering agents have a protective effect on high-risk patients with HTN. Although there is very little Korean data, a 50% reduction in low-density lipoprotein (LDL) cholesterol in patients who had an LDL cholesterol level ≥130 mg/dL significantly lowered the risk for CVD [94]. Lowering the LDL cholesterol level to <100 mg/dL is recommended in hypertension patients with coronary artery disease or diabetes mellitus [95]. For hypertension patients with acute coronary syndrome, LDL should be lowered below 70 mg/dL. There is evidence for reducing the LDL cholesterol level to <135 mg/dL in patients with stroke [96]; however, there is little data regarding the effect of lowering the LDL cholesterol to <70 mg/dL in such patients.

### Glycemic control

Recent clinical studies have shown that aggressive blood glucose control (a hemoglobin A1C level less than 6.0% or 6.5%) reduces the incidence of ischemic heart disease in patients with type 2 diabetes; however, there was no significant change or even increase in stroke or total mortality. Therefore, in patients with diabetes, the blood glucose level should be controlled so that the hemoglobin A1C is less than 7.0% in most patients and less aggressively (target hemoglobin A1C of 7.5% to 8.0%) in older patients or patients with diabetes of long duration, who have increased risk for hypoglycemia [97].

## Monitoring and follow-up

Patients should generally be followed up once monthly, at least until the target BP is achieved. Patients with severe HTN (stage two or more) need more frequent follow-up. The serum potassium and creatinine levels should be measured at least one to two times yearly. If the BP is controlled and stable, then the patient should be followed up every 3 to 6 months. A longer follow-up interval may be associated with low compliance. Therefore, patient compliance also must be monitored, and the need for blood tests should be emphasized to encourage attendance at follow-up visits. A longer follow-up interval to monitor the status of BP control can be achieved by encouraging home BP measurement.

## Compliance

Trust between doctor and patient is the most important issue in the treatment of HTN, and the patient should therefore be encouraged to participate in the development of the treatment plan. Many patients may have obtained information about various antihypertensive agents through various routes, so discussion may be necessary. First, identify the patient’s point of view to determine the relative importance of efficacy, cost-effectiveness, and side effects. It is necessary to reduce overall CV risk as much as possible while maintaining the patient’s compliance. Self-measurement of BP by using home BP monitoring can improve compliance.

## Abbreviations

ACE: Angiotensin-converting enzyme
ARB: Angiotensin receptor blocker
BMI: Body mass index
BP: Blood pressure
CKD: Chronic kidney disease
CV: Cardiovascular
CVD: Cardiovascular diseases
DBP: Diastolic blood pressure
DM: Diabetes mellitus
HDL: High-density lipoprotein
HTN: Hypertension
KNHANES: Korean National Health and Nutrition Examination Survey
LDL: Low-density lipoprotein
LVH: Left ventricular hypertrophy
SBP: Systolic blood pressure
